# Long term follow-up of persistent choroidal folds and hyperopic shift after complete removal of a retrobulbar mass

**DOI:** 10.1186/s13104-015-1610-1

**Published:** 2015-11-14

**Authors:** Agnes Galbo Jacobsen, Peter Bjerre Toft, Jan Ulrik Prause, Henrik Vorum, János Hargitai

**Affiliations:** Department of Ophthalmology, Aalborg University Hospital, Aalborg, Denmark; Eye Clinic, Rigshospitalet and Glostrup Hospital, University of Copenhagen, Copenhagen, Denmark; Eye Pathology Institute, Faculty of Health Sciences, University of Copenhagen, Copenhagen, Denmark; Department of Ophthalmology, Thy-Mors Hospital, Thisted, Denmark

**Keywords:** Choroidal fold, Hyperopic shift, Intraorbital mass, Cavernous haemangioma, Optical coherence tomography

## Abstract

**Background:**

Hyperopic shift and chorioretinal folds are common findings with intraorbital masses compressing the posterior pole of the globe. These signs usually regress after complete tumour excision. To the best of our knowledge this is the first reported case, where optical coherence tomography was used to document persistent chorioretinal folds after complete excision of a retrobulbar mass.

**Case presentation:**

A 47-year-old Caucasian woman was referred to our department with long-documented hyperopic shift and gradually decreasing vision in her left eye. Optical coherence tomography showed chorioretinal folds. Magnetic resonance imaging revealed a retrobulbar mass which caused flattening of the posterior pole of the globe. The tumour was successfully removed, and was confirmed to be a cavernous haemangioma on histological assessment. 3 years after surgery the patient still has a similar amount of hyperopia and chorioretinal folds.

**Conclusion:**

Choroidal folds and hyperopic shift may persist after complete tumour removal. Long term follow-up is advised to rule out recurrence of the intraorbital mass.

## Background

Cavernous haemangioma is the most common benign orbital neoplasm in adults [1]. Cavernous haemangiomas and other orbital tumours may compress the globe and induce choroidal folds and refractive changes. Choroidal folds are parallel grooves or striae involving the inner choroid, the Bruch’s membrane and the retinal pigment epithelium (RPE), and sometimes the retina (chorioretinal folds) [2]. Optical coherence tomography (OCT) provides cross-sectional images of the retina and choroid, making it useful for investigation of choroidal folds [3]. Symptoms from choroidal folds can vary depending on their cause and the rapidity of their progression. If the folds occur acutely, they can produce metamorphopsia caused by distortion of photoreceptors, but if they develop slowly vision can be preserved [3]. Hyperopic shift is a common finding if a mass compresses the posterior pole. Acquired chorioretinal folds and hyperopia usually regress after successful tumour removal [1, 4, 5]. We report a case with persistent hyperopia and chorioretinal folds 3 years after successful tumour removal.

## Case report

A 47-year-old Caucasian woman was referred to our department because of visual loss and left papillary oedema. She had originally contacted her ophthalmologist for heavy eyelids and a wish for upper blepharoplasty.

The patient history revealed fluctuating vision in the left eye for the previous 5 years and intermittent tinnitus in the left ear. The fluctuating vision was confirmed in notes obtained from her optometrist (Table 1). Apart from a hemithyroidectomy due to a “cold notch”, her past medical history was uneventful.

**Table 1:** Refraction and best-corrected visual acuity

Date	Ref. RE	BCVA RE	Ref. LE	BCVA LE
12/1999	+1.25D/−0.75 × 180	6/6	+1.0D/−0.5 × 180	6/6
8/2007	+2.5D/−0.5 × 5	6/6	+4.0D/−0.5 × 180	6/7.5
12/2009	+2.75D/−0.25 × 5	6/6	+5.75D/−0.25 × 180	6/10
03/2010	+2.75D/−1.0 × 10	6/6	+5.5D/−0.5 × 5	6/7.5
11/2010	+2.75D/−0.75 5	6/6	+5.75D/−0.5 × 5	6/6
*11/2011*	*+2.5D/−0.5* *×* *20*	*6/6*	*+6.5D/−1.25* *×* *0*	*6/12*
10/2012	+2.5D/−0.5 × 15	6/6	+6.0D/−1.25 × 0	6/9
09/2013	+2.5D/−0.5 × 15	6/6	+6.0D/−1.25 × 0	6/9
10/2014	+2.5D/−0.5 × 15	6/6	+6.0D/−1.25 × 0	6/8

A 15-year-long follow-up of the patient’s refraction and best-corrected visual acuity

Time of presentation (1 week prior to tumour removal) is highlighted in italics

*Ref* refraction, *BCVA* best-corrected visual acuity, *RE* right eye, *LE* left eye, *D* diopter

Refraction was +2.5D (diopter)/−0.5D × 20 in the right eye and +6.5D/−1.25D X 0 in the left eye. Best-corrected visual acuity (BCVA) was 6/6 in the right and 6/12 in left eye.

**Fig. 1:**
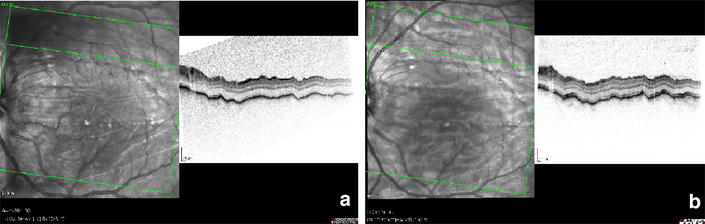
Optical coherence tomography imaging of the macula. Spectral-domain optical coherence tomography imaging showing chorioretinal folds of the left macula at presentation (**a**); unchanged folds 3 years after complete tumour removal (**b**)

The patient had 3 mm left axial proptosis measured with Hertel exophthalmometer. Full extraocular movements and no afferent pupillary defects were found. Funduscopy revealed horizontal chorioretinal folds and mild papilloedema. These findings were documented with spectral-domain optical coherence tomography (Fig. 1a).

**Fig. 2:**
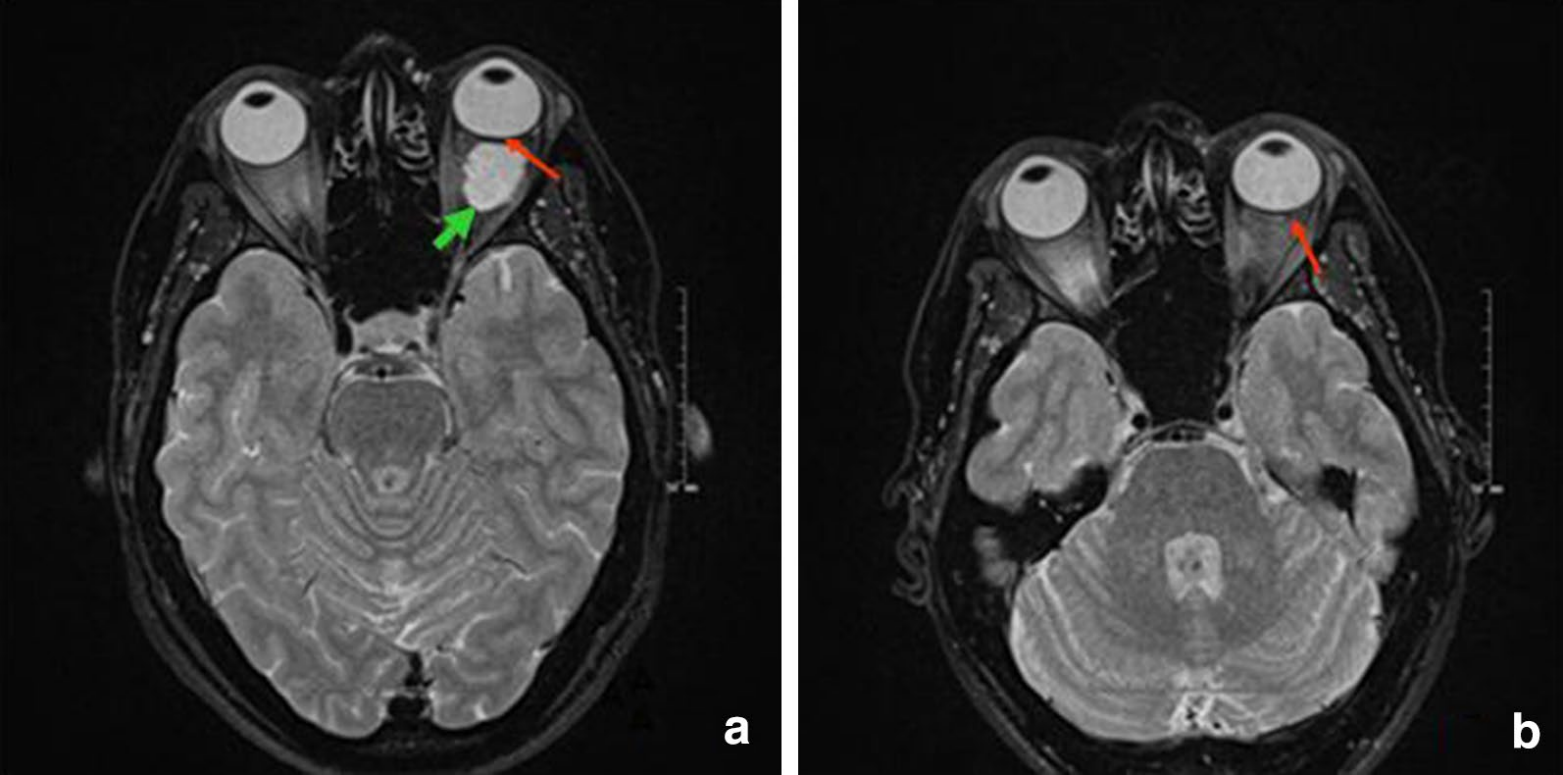
Magnetic resonance imaging of the orbit. Magnetic resonance imaging (T2), axial view showing the patient at presentation (**a**) with intraconal mass in the left orbit (*green arrow*), and flattening of the posterior pole (*red arrow*); 3 years after complete removal of the tumour, posterior pole flattening (*red arrow*) persisted (**b**)

Magnetic resonance imaging (MRI) showed a well-circumscribed, homogenous, 18 × 13.6 mm large, oval-shaped intraconal mass between the lateral rectus muscle and the optic nerve in the left orbit (Fig. 2a). A pronounced flattening of the posterior pole of the left globe was documented. Axial length (AXL) was 22.20 mm in the right eye and 20.80 mm in the left eye.

**Fig. 3:**
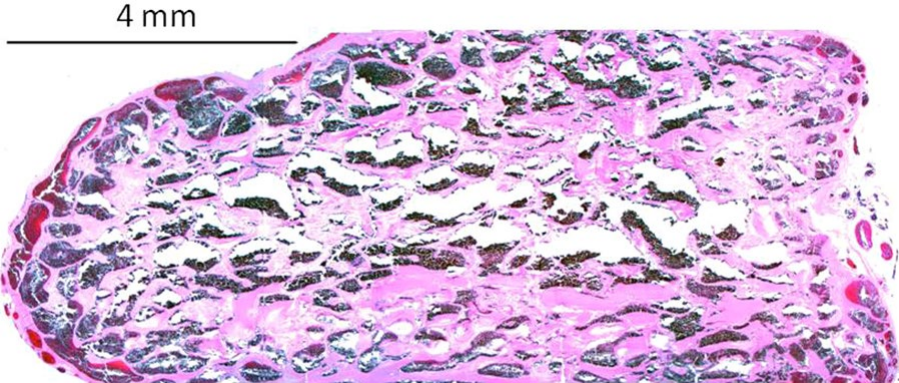
Histological examination. Histopathology of the excised tumour showing characteristic closely-spaced, thin-walled vessels. Hematoxylin and eosin staining

The patient was admitted to the university department where the tumour was removed through anterior orbitotomy. Histopathology confirmed a cavernous haemangioma of the orbit (Fig. 3). The patient made an uncomplicated recovery. She was followed every half year for 3 years after surgery. At the third-year control visit BCVA had improved to 6/8, and the refraction had stabilized at +6.0D/−0.75D X 175 in the left eye. Control OCT examination showed unchanged chorioretinal folds (Fig. 1b). Follow-up MRI showed no signs of recurrence, but the posterior flattening of the left globe remained unchanged (Fig. 2b). Control assessment found that AXL was 22.20 mm in right eye and 20.81 mm in the left eye.

## Conclusion

Several reports have confirmed that choroidal folds tend to regress after removal of an orbital mass [1, 4, 5]. However, in a few cases the folds persist for several years postoperatively [6]. The persistent flattening of the posterior pole and choroidal folds has been attributed to scleral remodelling after long-standing compression [6]. The presence of persisting flat posterior pole, chorioretinal folds, and hyperopia in our case may also be explained by this. Cavernous haemangiomas have a slow growth pattern; many patients have manifestation of tumour growth for more than 10 years without developing uncorrectable visual loss [7, 8]. Our patient had presumably developed the orbital mass several years before its diagnosis, which is supported by the longstanding visual symptoms; thus, an irreversible structural change of the sclera could have occurred. To the best our knowledge, this is the first reported case to show persistent chorioretinal folds and globe flattening after removal of a cavernous haemangioma of the orbit, confirmed by OCT and MRI.

## Abbreviations

BCVA: best-corrected visual acuity
D: diopter
MRI: magnetic resonance imaging
OCT: optical coherence tomography
RPE: retinal pigment epithelium
